# Apoptosis-mediated cytotoxic effects of parthenolide and the new synthetic analog MZ-6 on two breast cancer cell lines

**DOI:** 10.1007/s11033-012-2215-6

**Published:** 2012-10-14

**Authors:** Anna Wyrębska, Jacek Szymański, Katarzyna Gach, Justyna Piekielna, Jacek Koszuk, Tomasz Janecki, Anna Janecka

**Affiliations:** 1 Department of Biomolecular Chemistry, Medical University of Lodz, Mazowiecka 6/8, 92-215 Lodz, Poland; 2 Central Laboratory, Medical University of Lodz, Lodz, Poland; 3 Institute of Organic Chemistry, Technical University of Lodz, Lodz, Poland

**Keywords:** Breast cancer, α-Methylene-γ-lactones, Apoptosis, Cell cycle arrest

## Abstract

The search for effective plant-derived anti-cancer agents or their synthetic analogs has continued to gain interest in drug development. The anti-cancer activity of parthenolide (PTL) isolated from *Tanacetum parthenium*, has been attributed to the presence of α-methylene-γ-lactone skeleton. In the present study we aimed to investigate the anti-cancer potential of a new synthetic compound, 3-isopropyl-2-methyl-4-methyleneisoxazolidin-5-one (MZ-6), with the same as in PTL α-methylene-γ-lactone motif, on two breast cancer cell lines, MCF-7 and MDA-MB-231. For comparison, PTL was included in the study. PTL and MZ-6 reduced the number of viable MCF-7 and MDA-MB-231 cells, with half maximal inhibitory concentration values between 6 and 9 μM. Both compounds dose-dependently inhibited incorporation of [^3^H]thymidine, up-regulated Bax and down regulated Bcl-2 mRNA. The levels of the end product of lipid peroxidation, malondialdehyde, were significantly higher. In MCF-7 cells, MZ-6 induced early apoptosis and cell cycle arrest in G0/G1 phase. The effect produced by MZ-6 was much stronger compared with PTL. In MDA-MB-231 cells, both tested compounds had similar effect and induced mostly late apoptosis. In conclusion, the observed anticancer activity makes MZ-6 an attractive drug candidate and shows that simple analogs of α-methylene-γ-lactones can be good substitutes for more complex structures isolated from plants.

## Introduction

Breast cancer is one of the leading causes of mortality among women in developed countries. Approximately 60 % of breast cancers are hormone-dependent and require estrogen for tumor growth [1]. Recent progress in diagnosis and therapy has increased the survival of women with estrogen-dependent breast cancer. However, the treatment options available for estrogen-independent tumors are far from satisfactory.

In recent years the search for new anti-tumor substances obtained from plants has been a hot research activity. Among others, the sesquiterpene lactone, parthenolide (PTL), isolated from feverfew (*Tanacetum parthenium*) has been extensively studied in relation to its anti-cancer properties. PTL was shown in vitro to inhibit proliferation and induce apoptosis in various human cancers, such as colorectal cancer, hepatoma, cholangiocarcinoma and pancreatic cancer [2–5], but there have been no reports on its ability to induce apoptosis in breast cancer cells. PTL-induced apoptosis is associated with inhibition of transcription factor nuclear factor-κappa B (NF-κB) [6, 7], mitochondrial dysfunction and increase of reactive oxygen species [8]. The active fragment in the structure of PTL is an α-methylene-γ-lactone skeleton which can react via the Michael type addition with mercapto groups of enzymes and other functional proteins, interfering with key biological processes in the cell [9].

In our search for new anti-cancer agents, we have concentrated on the synthesis of simple heterocycles with the same as in PTL α-methylene-γ-lactone motif. In the previous paper [10] we have reported the synthesis of 3-isopropyl-2-methyl-4-methyleneisoxazolidin-5-one, designated MZ-6, with α-methylene-γ-lactone ring additionally modified by introduction of a nitrogen atom. Here, we tested the potential of MZ-6 to inhibit proliferation and induce apoptosis in estrogen-dependent, minimally invasive and estrogen-independent, highly invasive breast cancer cell lines, MCF-7 and MDA-MB-231, respectively. For comparison, PTL was included in the study.

## Materials and methods

### Materials and general procedures

**Fig. 1:**
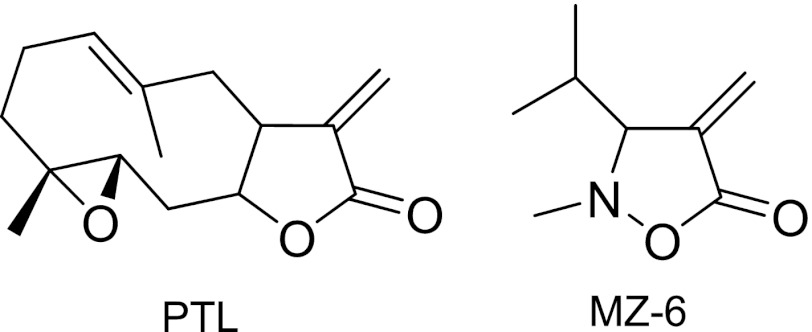
Structure of parthenolide (PTL) and a synthetic compound, 3-isopropyl-2-methyl-4-methyleneisoxazolidin-5-one (MZ-6), with the same as in PTL α-methylene-γ-lactone motif, additionally modified by introduction of an nitrogen atom

PTL was purchased from Tocris Bioscience (Bristol, UK). 3-Isopropyl-2-methyl-4-methyleneisoxazolidin-5-one (MZ-6) was synthesized in a two step reaction sequence, published earlier [10]. Briefly, 2-diethoxyphosphoryl-4-methyl-2-pentanoic acid in the reaction with N-methylhydroxyloamine hydrochloride yielded 4-diethoxyphosphoryl-3-isopropyl-2-methylisoxazolidin-5-one which was used as a Horner-Wadsworth-Emmons reagent in the olefination of formaldehyde to give the target compound. The purity of MZ-6 was confirmed by ^1^H and ^13^C NMR and elemental analysis [10]. The structures of PTL and MZ-6 are shown in Fig. 1.

For all experiments PTL and MZ-6 were dissolved in DMSO and further diluted in distilled water to obtain 0.1 % DMSO concentration. In each experiment controls without and with 0.1 % DMSO were performed. 0.1 % DMSO had no effects on the observed parameters.

### Cell culture

The MCF-7 and MDA-MB-231 cell lines were purchased from the European Collection of Cell Cultures (ECACC). The MCF-7 cells were cultured in Minimum Essential Medium Eagle (MEME, Sigma-Aldrich, St. Louis, MO, USA) with glutamine (2 mM) (Sigma-Aldrich, St. Louis, MO, USA), Men Non-essential amino acid solution (1 %) (Sigma-Aldrich, St. Louis, MO, USA), gentamycin (5 μg/mL) and 10 % heat-inactivated fetal bovine serum (FBS) (both from Biological Industries, Beit-Haemek, Israel). The MDA-MB-231 cells were cultured in high glucose Dulbecco’s Modified Eagle Medium (DMEM, BioWhittaker, Lonza, Basel, Switzerland) supplemented with glutamine (2 mM) (Sigma-Aldrich, St. Louis, MO, USA) gentamycin (5 μg/mL) and 10 % heat-inactivated fetal bovine serum (FBS) (both from Biological Industries, Beit-Haemek, Israel). Cells were maintained at 37 °C in a 5 % CO_2_ atmosphere and were grown until 80 % confluent.

### Cell viability assay (MTT)

Cell viability was determined by the mitochondrial reduction assay (MTT), as described elsewhere [11]. MCF-7 or MDA-MB-231 cells were grown to subconfluent levels in MEME or DMEM, respectively, with 10 % FBS and then plated onto 24-well culture plates (10^4^ cells/well) in the final volume of 1 mL of culture medium. After 24 h, various concentrations of MZ-6 or PTL were added and the plates were incubated for 48 h. Afterwards, cells were incubated for 2 h at 37 °C with 3-(4,5-dimethylthiazol-2-yl)-2,5-diphenyltetrazolium bromide (MTT, 5 mg/mL in PBS) and the metabolically active cells reduced the dye to blue formazan product. The absorbance was measured at 540 nm and compared with control (untreated cells). All experiments were performed in triplicate.

### Sulphorhodamine (SRB) cytotoxicity assay

MCF-7 and MDA MB 231 cells were seeded in the 96-well plates at the density 6 × 10^3^ and 3 × 10^3^ cells/well, respectively, as described earlier [12]. After 24 h, various concentrations of MZ-6 or PTL were added and the plates were incubated for 48 h. Then cells were fixed with ice-cold 10 % trichloroacetic acid solution, by gently placing the solution on top of the cells. After 60 min at 4 °C, cells were washed five times with distilled and de-ionized water and air dried. Then 50 μL of 0.4 % SRB solution in 1 % acetic acid were added to the cells, followed by 20 min incubation. The cells were washed five times with 1 % acetic acid solution and again air dried, 100 μL of 10 mM Tris solution (pH 10) was added to dissolve the bound dye and plates were placed on a plate shaker for 10 min at room temperature. The absorbance was measured at 490 nm using an authomated plate reader (iMark Bio-Rad, Hercules, CA, USA) and compared with controls (untreated cells). All experiments were performed in triplicate.

### [^3^H]Thymidine incorporation assay

The MCF-7 and MDA-MB-231 cells were seeded in the 6-well plates at a density 0.3 × 10^6^ and 0.2 × 10^6^ cells/well, respectively, in 3 mL of cell growth medium. After 24 h cell growth medium was replaced with 2 mL serum free MEME (for MCF-7) or DMEM (for MDA-MB-231) growth medium. On the third day various concentrations of MZ-6 or PTL were added and the plates were incubated for 24 h. [^3^H] thymidine (2 μCi/well) was added and after 7 h cells were washed with trichloroacetic acid and then lysed by NaOH. [^3^H]thymidine incorporated into DNA was measured using a scintilation counter (Packard Tri-Carb 2100 TR, Ramsey, MN, USA) [13].

### Cell morphology assessment

Confluent cultures of MCF-7 and MDA-MB-231 cells were treated with the tested compounds for 0, 6, 12 and 24 h and photographed at the reference points with a phase-contrast microscope (OLYMPUS CKX41, Olympus, London, UK) (magnification 200x).

### Quantitative real-time PCR assay

The MCF-7 or MDA-MB-231 breast cancer cells were seeded in 25 cm^2^ cell culture flasks in 10 mL of standard growth medium. After 24 h, the growth medium was replaced by a fresh growth medium supplemented with the tested compounds in the desired concentrations. The cells incubated without tested compounds were used as the controls. After 24 h of incubation, the cells were washed twice with phosphate buffered saline (PBS, GIBCO, Invitrogen, Carlsbad, CA, USA) to remove added compounds and harvested by trypsinization. The cells were frozen and kept at −80 °C awaiting further experiments.

Total RNA was extracted from the MCF-7 or MDA-MB-231 cells using Total RNA Mini Kit (A&A Biotechnology, Poland) according to the manufacturer’s protocol. The concentration and purity of isolated RNA were determined spectrophotometrically at 260 and 280 nm. cDNA was synthesized using Enhanced Avian HS RT-PCR Kit and oligo(dT)_12-18_ primers (Sigma-Aldrich, St. Louis, MO, USA). The expression of Bax and Bcl-2 genes, as well as glyceraldehyde 3-phosphate dehydrogenase (GAPDH), used as a house-keeping gene, was quantified by real-time PCR using an Mx3005P QPCR Systems (Agilent Technologies, Inc. Santa Clara, CA, USA) according to the manufacturer’s instructions for the Brilliant II SYBR Green QPCR Master Mix (Agilent Technologies, Inc. Santa Clara, CA, USA). Primers used are listed in Table 1.

**Table 1:** Primers used for quantitative real-time PCR

Target gene	Forward primers	Reverse primers
Bax	5′ ACCCGGTGCCTCAGGATGCGT 3′	5′ GGCAAAGTAGAAAAGGGCGAC 3′
Bcl-2	5′ CATGCTGGGGCCGTACAG 3′	5′ GAACCGGCACCTGCACAC 3′
GAPDH	5′ GTCGCTGTTGAAGTCAGAGGAG 3′	5′ CGTGTCAGT GGTGGACCTGAC 3′

Real-time PCR reactions were run in triplicate using the following thermal cycling profile: 95 °C for 10 min, followed by 40 steps of 95 °C for 30 s and 58 °C for 1 min and 72 °C for 1 min. After 40 cycles, samples were run for the dissociation protocol (i.e. melting curve analysis). Brilliant II SYBR Green fluorescence emissions were captured and mRNA levels were quantified using the critical threshold (Ct) value. Controls without reverse transcription and with no cDNA template were included with each assay. Relative gene expression levels were obtained using the ∆∆Ct method [14]. All results are presented as mean ± SD.

### Analysis of apoptosis by flow cytometry

Apoptotic cell death was determined using the FITC Annexin V Apoptosis Detection Kit (BD Bioscience), according to the manufacturer guidelines. Briefly, MCF-7 and MDA-MB-231 cells were treated for 24 h with the indicated concentrations of the tested compounds. Then cells were harvested, stained with annexin V and propidium iodide (PI) and kept for 15 min at room temperature in the dark. Apoptosis of cells was analyzed by flow cytometry (Becton–Dickinson Canto II). Early and late apoptosis was visualized and quantified by constructing a dot-plot using BD FACSDiva software.

### Cell cycle analysis by flow cytometry

Cells were seeded in 25 cm^2^ flasks and incubated overnight to allow cells to attach to the plate. After treatment of the cells with indicated concentrations of the tested compounds (or DMSO as control) for 24 h, cells were harvested, washed twice with PBS and fixed in 70 % ethanol. Immediately before the analysis, the cells were washed once with PBS and re-suspended in PI/RNase staining buffer (BD Bioscience). The cell suspensions were incubated for 15 min at room temperature. The distribution of cells in the cell cycle was measured by flow cytometry. Percentages of cells in the cell cycle phases were calculated using ModFit LT 3.3 software.

### Lipid peroxidation assay

Lipid peroxidation was performed using thiobarbituric acid-reactive substances (TBARS) protocol [15]. Briefly, cells incubated for 24 h with a tested compound or control cells were collected by centrifugation, sonicated in ice-cold potassium chloride (1.15 %) and again centrifuged for 10 min. The resulting supernatant (0.5 mL) was added to 0.5 mL of 15 % trichloroacetic acid (TCA) in 0.25 M HCl and 0.5 mL of 0.37 % of thiobarbituric acid (TBA) in 0.25 M HCl and heated at 100 °C for 15 min in a boiling water bath. Then the samples were cooled and centrifuged for 10 min. Absorbance of the supernatants was measured at 535 nm. Data were expressed as percent of the control.

### Statistical analysis

Statistical analyses were performed using Prism 4.0 (GraphPad Software Inc., San Diego, CA, USA). The data were expressed as means ± SD. Differences between groups were assessed by a one-way ANOVA followed by a post hoc multiple comparison Student–Newman–Keuls test. *p < 0.05, **p < 0.01 ***p < 0.001 was considered as significantly different from untreated cells regarded as control.

## Results

### Cytotoxic activity

The cytotoxic effect of PTL and MZ-6 on MCF-7 and MDA-MB-231 breast cancer cells was determined using two assays, the MTT and SRB assay. The principle of MTT assay is the cellular reduction of MTT by the mitochondrial dehydrogenase of viable cells. A blue formazan that is formed can be measured spectrophotometrically. The SRB is a colorimetric assay measuring the uptake of the negatively charged pink aminoxanthine dye, sulphorhodamine B, by basic amino acids in functional lysosomes. The survival of MCF-7 and MDA-MB-231 cells following incubation with increasing concentrations of the tested compounds for 48 h was assessed. Proliferation of both types of cells was inhibited by PTL and MZ-6 with IC_50_ values ranging from 3 to 12 μM (Table 2). Small differences between the IC_50_ values obtained by MTT and SRB assay were observed. The values obtained using MTT were usually slightly lower. In the following experiments in which the tested compounds were used at the IC_50_ concentrations, the mean values of both assays were used.

**Table 2:** Inhibition of proliferation of MCF-7 and MDA-MB-231 cells by PTL and MZ-6

Compound	IC_50_^a^ (μM)					
	MCF-7			MDA-MB-231		
	MTT	SRB	Mean value^b^	MTT	SRB	Mean value^b^
PTL	9.5 ± 0.70	9.5 ± 0.75	9.5	8.0 ± 0.50	7.4 ± 0.32	7.7
MZ-6	4.5 ± 0.70	10 ± 0.49	7.25	3.5 ± 0.60	9.5 ± 0.40	6.5

^a^IC_50_ values represent 50 % inhibitory concentration of the tested compound. Data are mean ± SD of three independent experiments

^b^IC_50_ concentration used in experiments

### [^3^H]Thymidine incorporation assay

**Fig. 2:**
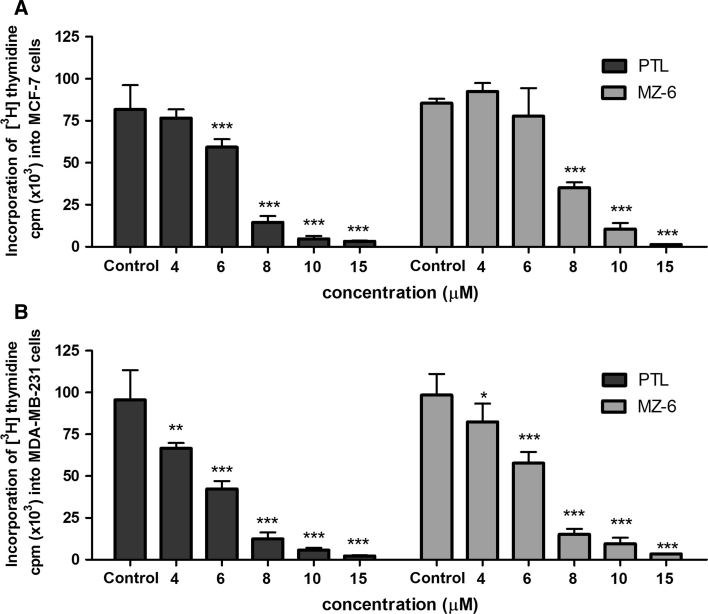
Incorporation of [^3^H]thymidine into **A** MCF-7, **B** MDA-MB-231 cells treated for 24 h with PTL or MZ-6 at IC_50_ concentrations. Data represent mean ± SD of three independent experiments. Statistical significance was assessed using one-way ANOVA and a post hoc multiple comparison Student–Newman–Keuls test. *p < 0.05, **p < 0.01, ***p < 0.001 was considered as significantly different from untreated cells regarded as control

[^3^H]Thymidine incorporation into DNA was used as an index of cell proliferation. This assay allows the assessment of the level of incorporated radiolabeled thymidine into DNA during S phase of the cell cycle, as a quantitative measure of new DNA synthesis. As shown in Fig. 2A, B, the concentration-dependent inhibition of cell proliferation following 24 h incubation with PTL or MZ-6 was observed in both types of cells. No significant differences between cell lines and tested compounds were found.

### Assessment of changes in cell morphology

The ability of PTL and MZ-6 to induce apoptosis was first evaluated based on the changes in cell morphology. The most recognizable morphological changes observed under the phase-contrast microscope included the cytoplasmic condensation resulting in cell shrinkage caused by loss of water, cytoskeletal disintegration and loss of contact with the base (data not shown).

### Assessment of changes in gene expression on the mRNA level

**Fig. 3:**
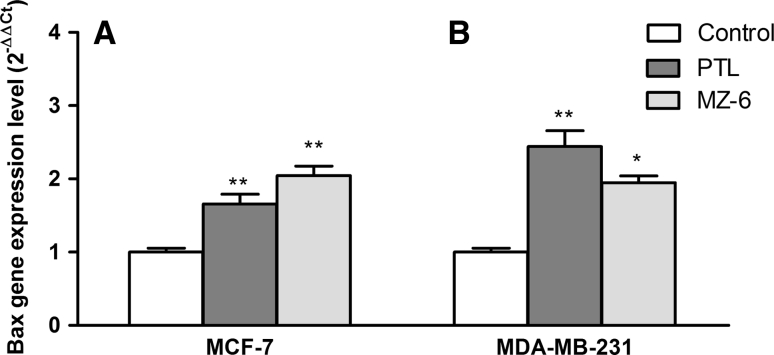
Quantitative real-time PCR analysis of Bax mRNA levels in: **A** MCF-7 and **B** MDA-MB-231 cells incubated for 24 h with PTL or MZ-6 at IC_50_ concentrations. Data represent mean ± SD of three independent experiments. Statistical significance was assessed using one-way ANOVA and a post hoc multiple comparison Student–Newman–Keuls test. *p < 0.05, **p < 0.01, was considered as significantly different from untreated cells regarded as control

**Fig. 4:**
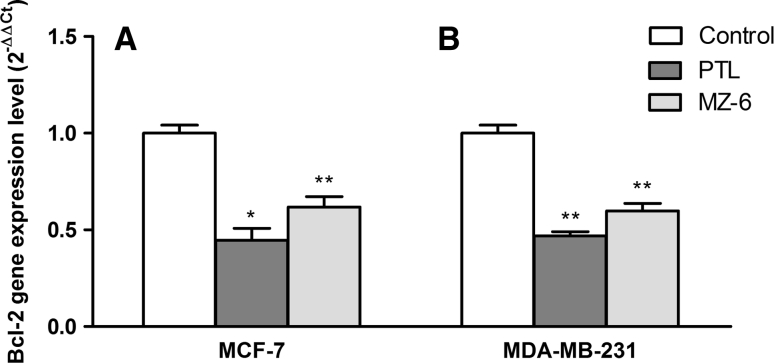
Quantitative real-time PCR analysis of Bcl-2 mRNA levels in: **A** MCF-7 and **B** MDA-MB-231 cells incubated for 24 h with PTL or MZ-6 at IC_50_ concentrations. Data represent mean ± SD of three independent experiments. Statistical significance was assessed using one-way ANOVA and a post hoc multiple comparison Student–Newman–Keuls test. *p < 0.05, **p < 0.01, was considered as significantly different from untreated cells regarded as control

Many drugs exert their cytotoxic effect by triggering the intrinsic apoptotic pathway [16]. Since Bcl-2 family of proteins are important factors in regulation of apoptosis, we examined changes in the gene expression of pro-apoptotic (Bax) and anti-apoptotic (Bcl-2) proteins in MCF-7 and MDA-MB-231 cells treated for 24 h with PTL or MZ-6 at the IC_50_ concentration each. The mRNA levels of these genes were determined by real-time PCR and compared with controls. The tested compounds up-regulated Bax mRNA levels in both cell lines. However, the effect produced by MZ-6 was stronger in MCF-7 cells, while PTL induced higher up-regulation of Bax in MDA-MB-231 cells (Fig. 3A, B). PTL and MZ-6, both down-regulated Bcl-2 to the similar degree (Fig. 4A, B).

### Analysis of apoptosis by flow cytometry

The combination of fluorescein isothiocyanate (FITC)-labeled annexin V (annexinV-FITC) with PI has been used to quantitatively determine the percentage of cells within a population that are actively undergoing apoptosis. In apoptotic cells, the membrane phospholipid, phosphatidylserine, is translocated from the inner to the outer portion of the membrane, and thus can be detected by annexin V-FITC, a phospholipid binding protein [17]. Meanwhile, the cells with disintegrated membrane can be detected by the binding of PI to their cellular DNA.

**Fig. 5:**
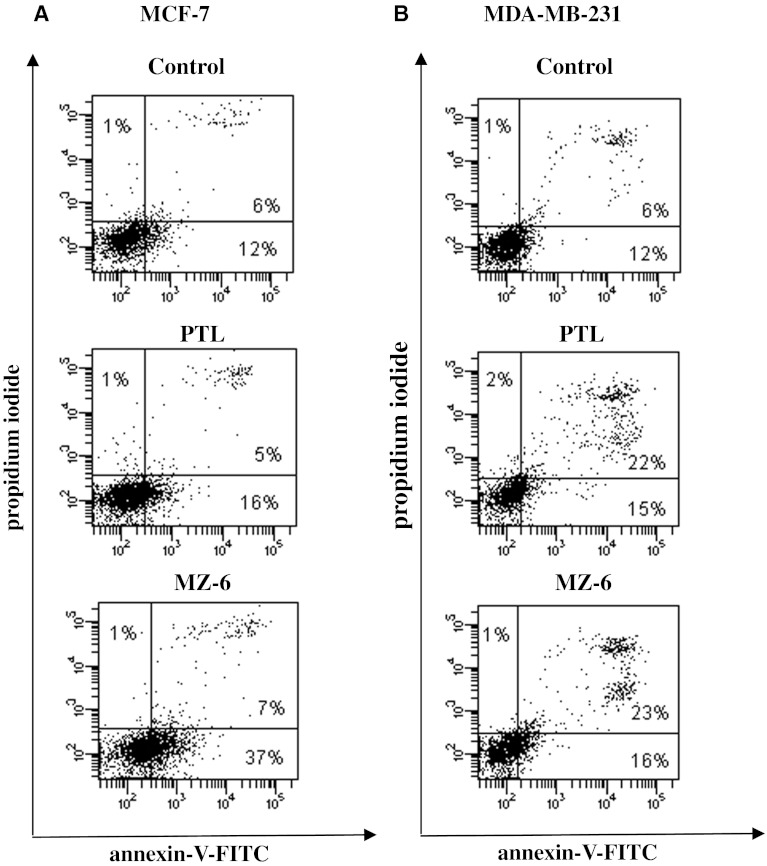
Effect of PTL and MZ-6 on the induction of apoptosis in: **A** MCF-7 and **B** MDA-MB-231 cells after 24 h treatment of cells with PTL or MZ-6 at IC_50_ concentrations. The cells were stained with FITC conjugated Annexin V in a buffer containing propidium iodide and analyzed by flow cytometry. For each group of cells the percentage of surviving cells is shown in the lower left quadrant. The lower right quadrant indicates the percentage of early apoptotic cells (Annexin V *positive cells*). The percentage of late apoptotic cells is shown in the upper right quadrant (Annexin V and PI *positive cells*)

To investigate the induction of apoptosis in MCF-7 and MDA-MB-231 cells, incubated for 24 h with PTL or MZ-6 at IC_50_ concentration, the flow cytometric analysis was performed. Annexin V/PI analysis was applied to quantify the apoptotic profile (Fig. 5A, B). Annexin V-positive cells (right, upper and lower quadrants in the density dot-plot) include early apoptotic (lower) and late apoptotic (upper) cells. The inductive effect of apoptosis was concentration dependent (data not shown). In MCF-7 cells both, PTL and MZ-6 increased the number of early apoptotic cells in the population up to 16 and 37 %, respectively, as compared with control (12 %). No changes were observed in the population of late apoptotic cells. In MDA-MB-231 cells PTL, as well as MZ-6, induced an increase of early (15 and 16 %, respectively) and also late apoptosis (22 and 23 %, respectively), compared with control (12 for early and 6 % for late apoptosis). In this cell line both tested lactones induced mainly late apoptosis and no statistically significant differences were observed between them.

### Analysis of cell cycle by flow cytometry

**Fig. 6:**
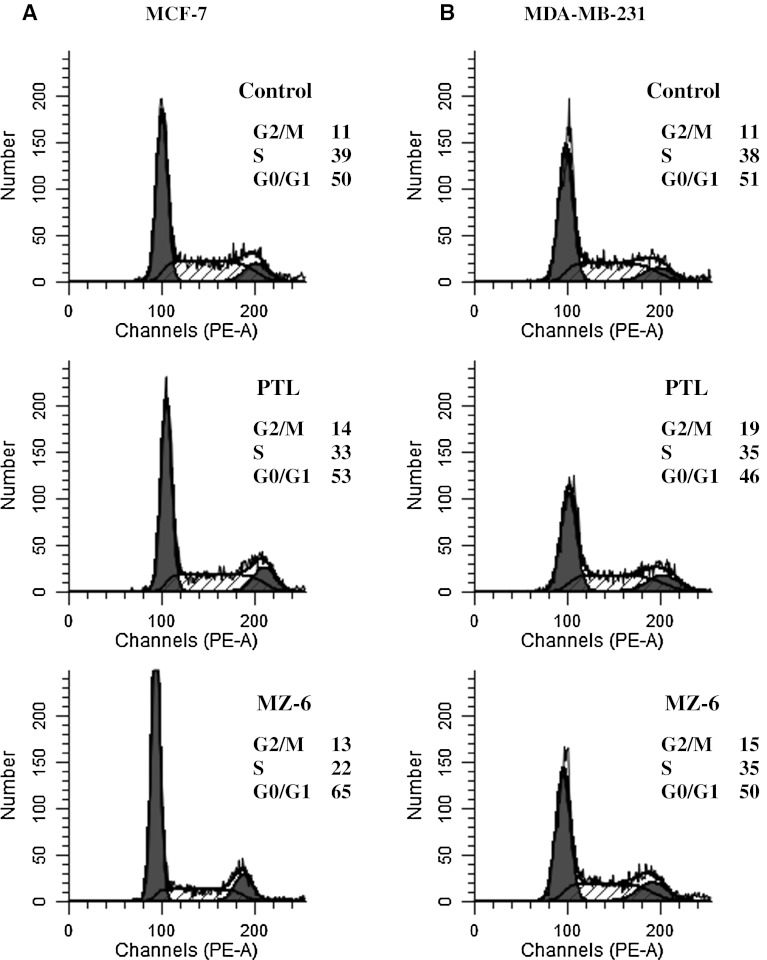
Effect of PTL and MZ-6 on cell cycle in: **A** MCF-7 and **B** MDA-MB-231 cells. Cells were cultured for 24 h with PTL or MZ-6 at IC_50_ concentrations. The percentage of cells at each stage of cell cycle was analyzed by flow cytometry, following DNA staining with propidium iodide and compared with control (*untreated cells*). The results are representative of three independent experiments

Cell cycle distribution in MCF-7 and MDA-MB-231 cells was studied after 24 h exposure to PTL or MZ-6 at IC_50_ concentration each (Fig. 6A, B). The stained DNA of MCF-7 cells treated with MZ-6 demonstrated that 65 % of the cells was in the G0/G1 phase, as compared to 50 % in control. This indicates that in MCF-7 cells MZ-6 caused G0/G1 cell cycle arrest. In MDA-MB-231 cells MZ-6 had no effect on the cell cycle. PTL in any of the two tested cell lines influenced the cell cycle.

### Lipid peroxidation

Imbalance between the production of reactive oxygen species (ROS) and the ability of the cell to detoxify these reactive intermediates leads to oxidative stress. The anti-cancer activity of PTL in several cell lines has been suggested to be due to its pro-apoptotic action which might occur through the increased production of ROS [18]. Because ROS have extremely short half-lives, they are difficult to measure directly. Instead, what can be measured are several products of the damage produced by oxidative stress, such as thiobarbituric acid reactive substances TBARS.

Assay of TBARS measures malondialdehyde (MDA) which is one of several low-molecular-weight substances formed via the decomposition of lipid peroxidation products. MDA reacts with TBA and the concentration of the resulting MDA-TBA adduct can be measured spectrophotometrically.

**Fig. 7:**
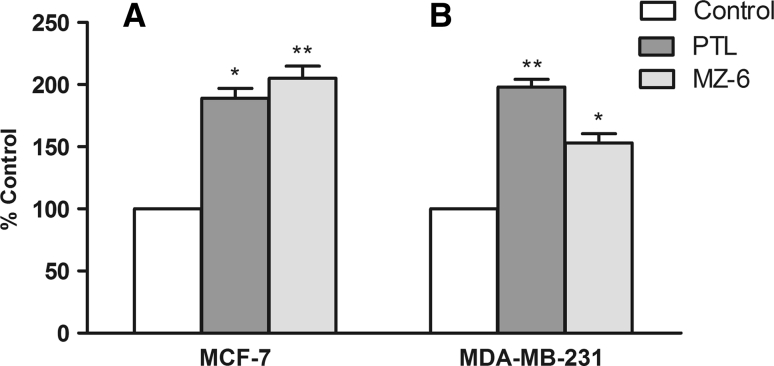
Lipid peroxidation measured as percentage of thiobarbituric acid reactive substances (TBARS) in: **A** MCF-7 and **B** MDA-MB-231 cells incubated for 24 h with PTL or MZ-6 at IC_50_ concentrations Data were expressed as percent of the control and represent mean ± SD of three independent experiments. Statistical significance was assessed using one-way ANOVA and a post hoc multiple comparison Student–Newman–Keuls test. *p < 0.05, **p < 0.01, was considered as significantly different from untreated cells regarded as control

We found that in MCF-7 and MDA-MB-231 cells treated for 24 h with MZ-6 or PTL at IC_50_ concentration, the levels of MDA, an end product of lipid peroxidation, were significantly higher than in control cells (Fig. 7A, B). In MCF-7 cells MZ-6 induced more than a 100 % increase of MDA level and was more active than PTL. In MDA-MB-231 cells PTL produced similar effect as in MCF-7 cells, while the increase of MDA level by MZ-6 in these cells was only about 60 % of the control.

## Discussion

Apoptosis is an active process of normal cell death in the development and maintenance of tissue homeostasis. Its morphological characteristics include plasma membrane blebbing, cell shrinkage, nuclear condensation, chromosomal DNA fragmentation, and formation of apoptotic bodies [19].

Breast epithelial cells undergo cyclic proliferation and apoptosis during the menstrual cycle [20]. One of the molecules responsible for maintaining the balance between cell proliferation and apoptosis is anti-apoptotic Bcl-2 protein, the expression of which also fluctuates in cyclic pattern in normal breast epithelium [16]. It has been reported that Bcl-2 protein family is associated with mitochondria-related apoptosis [21, 22]. Anti-apoptotic Bcl-2 protein is localized on the outer mitochondrial membranes and antagonizes the action of pro-apoptotic Bax. The balance between these two proteins prevents translocation of cytochrome c from the mitochondria and determines the apoptosis resistance. Inhibition of Bcl-2 or/and induction of Bax breaks this balance and leads to apoptosis. Tumor suppressor protein P53, which plays a role in DNA repair and cell cycle arrest, is also involved in apoptosis regulation. The expression of these apoptosis regulating factors, Bax/Bcl-2 and P53 is inter-related in the apoptosis of cancer cells, including breast cancer [23].

It was postulated earlier that compounds bearing α-methylene-γ-lactone motif can mediate their cytotoxic effect by triggering apoptosis [24–26]. In this study, we investigated the signalling pathways affected by a well-known sesquiterpene lactone, PTL and a simple synthetic compound with the same α-methylene-γ-lactone skeleton, MZ-6, in two breast cancer cell lines with varying invasiveness. MCF-7 and MDA-MB-231 represent, respectively, low and high invasive attributes of breast carcinoma. The obtained results demonstrated that both tested compounds inhibited proliferation of cells from both cell lines. Additionally, PTL as well as MZ-6 treatment led to a significant chromatin condensation, cell shrinkage and loss of contact with the base which pointed at the occurrence of apoptosis. To gain further insight into the way in which both tested lactones were cytotoxic, some key molecules known to regulate apoptosis were investigated. We have observed that the expression of pro-apoptotic Bax was up-regulated and the anti-apoptotic Bcl-2 was down-regulated, which is indicative of induction of apoptosis. However, MZ-6-induced up-regulation of Bax was more pronounced in MCF-7 cells, while the effect produced by PTL was stronger in MDA-MB-231 cells. Both lactones exerted similar down-regulation of Bcl-2.

Furthermore, apoptosis was examined by flow cytometry after combined staining with annexin V and PI, which is the most reliable and advanced method for detecting early apoptosis. Since the induction of apoptosis might be mediated through the cell cycle arrest, we next examined the effect of PTL and MZ-6 on cell cycle perturbations. Different results were obtained for both cell lines. In MCF-7 cells, MZ-6 induced early apoptosis and cell cycle arrest in G0/G1 phase. The effect produced by MZ-6 was much stronger compared with PTL. In MDA-MB-231 cells, both tested compounds induced mostly late apoptosis and had no influence on cell cycle. Thus, induction of apoptosis in both cell lines differed in time; in the same time period (24 h) MCF-7 cells were still in early apoptosis, while MDA-MB-231 cells in late apoptosis. This indicates that MDA-MB-231 cells are more sensitive to α-methylene-γ-lactones than MCF-7 cells. Induction of apoptosis was caused by disturbance of the redox status and resulting oxidative stress.

The differential responses between the two cell lines could be attributed to their different estrogen receptor status. However, these cell lines are also distinctive in *P53* and caspase-3 status, both of which are critical in cell cycle and apoptosis regulation. MDA-MB-231 cells express mutant *P53* and functional caspase-3 [27], whereas MCF-7 cells express wild type *P53* and are caspase-3 deficient [28]. It will be of great interest to further reveal molecular mechanisms underlying current observations.

## Conclusions

Anti-cancer activity and molecular mechanisms of action of various compounds with α-methylene-γ-lactone skeleton isolated from plants have been extensively studied, especially in the last decade. A number of purely synthetic compounds with the same structural motif has also been published but their biological evaluation was limited mostly to assessing their cytotoxic activity against selected cancer cell lines. In this study we compared pro-apoptotic activity of a new synthetic α-methylene-γ-lactone (MZ-6), which was additionally modified by introduction of a nitrogen atom, and a well-known natural product, PTL, on two breast cancer cell lines, MCF-7 and MDA-MB-231.

MZ-6 was shown to effectively inhibit proliferation and induce apoptosis in both types of breast cancer cells but had stronger effect on estrogen-dependent MCF-7 cells. Although the molecular mechanisms of MZ-6-induced effect on cancer cells still need to be explored in more detail, our data indicate that the changes in gene expression of Bcl-2 family of proteins, followed by induction of apoptosis and also cell cycle arrest were involved in the cytotoxic activity of this compound. The obtained results prove that simple synthetic analogs of α-methylene-γ-lactones can be good substitutes for more complex structures isolated from plants.
